# Lichen Planus Mimicking Atypical Melanocytic Lesion in a Man With Dark Skin

**DOI:** 10.5826/dpc.1003a60

**Published:** 2020-06-29

**Authors:** Federica Scarfì, Luciana Trane, Flavia Silvestri, Federico Venturi, Teresa Oranges, Agata Janowska, Francesca Portelli, Vincenzo De Giorgi

**Affiliations:** 1 Department of Dermatology, University of Florence, Florence, Italy; 2 Division of Pathological Anatomy, Department of Surgery and Translational Medicine, University of Florence, Florence, Italy; 3 Cancer Research Attilia Pofferi Foundation, Pistoia, Italy

**Keywords:** lichen planus, melanoma, dark skin, dermoscopy

## Introduction

Lichen planus is an idiopathic lichenoid dermatosis that has many variants, such as the follicular form, which is clinically and histologically characterized by follicle involvement. Usually the most typical dermoscopic feature of lichen planus is the presence of whitish lines, also called Wickham striae (WS), which histologically correlate with orthokeratosis above the zones of wedge-shaped hypergranulosis [1]. WS are seen as rounded, arboriform, reticular, or annular pearly whitish structures and are pathognomonic of lichen planus. WS can show different color patterns, such as homogeneous crystalline white, blue-white veil, and yellowish white pattern. The WS border can show thin spikes or arboriform ramifications, intermingled with linear vessels [1,2].

Other features that can be observed at dermoscopic analysis are the presence of gray-blue dots, peppering, red lines due to the presence of vascular structures, comedo, milium-like cysts, and blue-white veils in the center [2].

## Case Presentation

**Figure 1:**
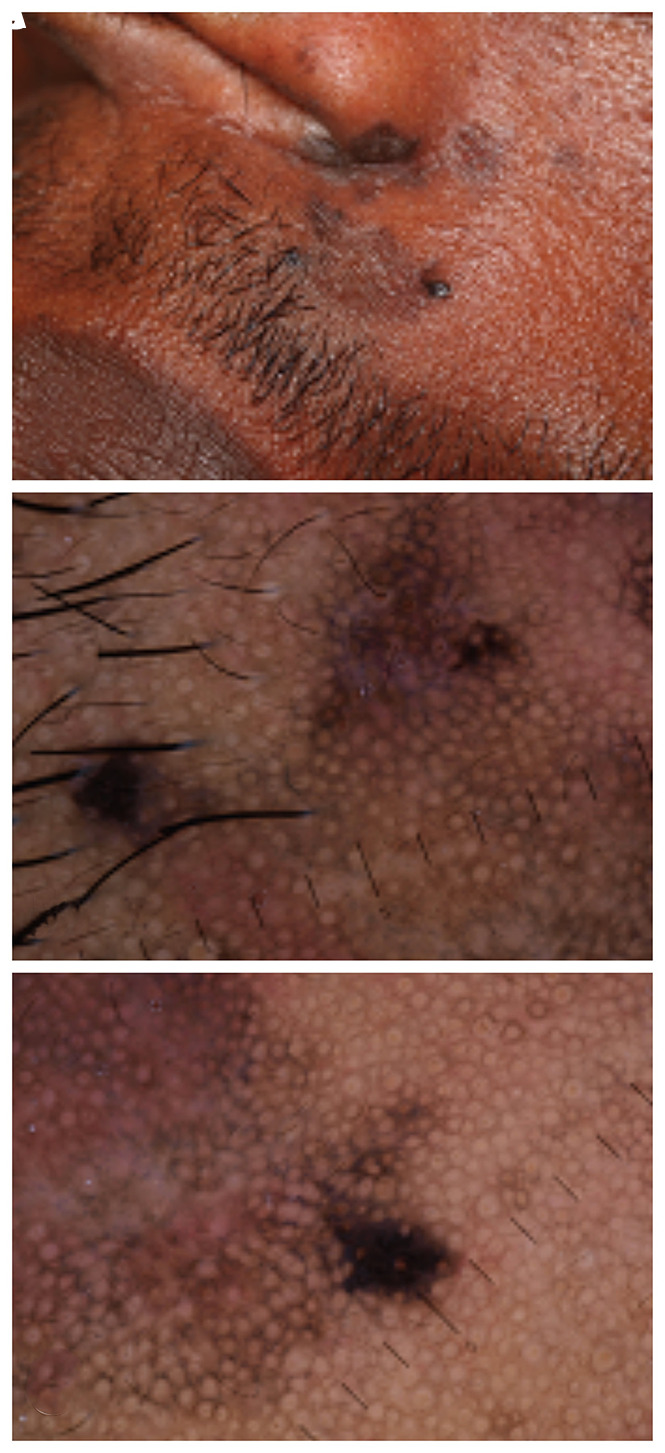
(A) Clinical and (B,C) dermoscopic features of the case.

A dark-skinned man of African origin, aged 33, who has been living in Italy for the past 10 years, came to our attention after a 1-year history of a pigmented lesion on his face. This lesion was previously treated as actinic keratosis with diclofenac sodium 3% gel, but this treatment had not been successful. On examination, the area of concern showed irregularly flat, dark brown macules with defined limits on nasolabial folds. The lesion showed 2 slightly elevated darker infiltrated and palpable areas, 1 on the left side of the upper lip and 1 under the nostril (Figure 1A). The patient did not report any symptoms.

Dermoscopy showed the typical pseudonetwork pattern with gray-brown perifollicular pigmentation, reticulated hyperpigmentation and 2 areas of darker irregular pigmentation, and obliteration of follicular openings (Figure 1B).

According to the dermoscopic features, the recent onset of the lesion, and its increased size, two 4-mm punch biopsies were taken for histopathological examination under the suspected diagnosis of atypical melanocytic lesion.

**Figure 2:**
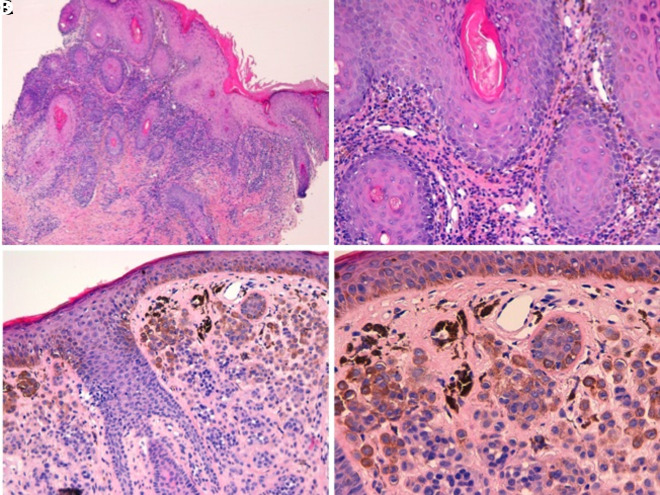
(A,B) Histopathological examination of 2 punch biopsies shows compact hyperkeratosis, irregular epidermal hyperplasia with vacuolar basal changes, and scattered apoptotic keratinocytes. There is a band-like inflammatory infiltrate in the dermis consisting of lymphocytes and histiocytes and scattered melanophages. Delicate fibroplasia around adnexal follicles is also observed. (C,D) The second skin biopsy shows a common dermal nevus, with pigmented melanocytes in the superficial portions of the lesion; melanocytes do not show signs of cytological atypia.

The final clinical and histopathological diagnosis (Figure 2) was follicular lichen planus with the presence of a dermal melanocytic nevus (Figure 1C).

## Conclusions

The particular site of the face and the dark skin of our patient can hide some typical features of follicular lichen planus and can mimic some typical features of melanocytic lesions. The pathognomonic WS may not be visible in pigmented skin and WS may not be appreciated on lichen planus lesions when the patient has been receiving topical treatment such as topical steroids or salicylic acid.

Dermoscopic analysis of our case showed a gray pseudonetwork with asymmetrical pigmented follicular openings. This pattern, however, is common to numerous pigmented lesions, both melanocytic and not melanocytic lesions, which often makes a correct diagnosis difficult.

Moreover, the presence of a milky pink erythema in a part of the lesion and the obliteration of some follicular openings can mean a progression of malignant lentigo [2].

Our case shows all typical dermoscopic features of a melanocytic lesion on the face. Therefore, the preoperative clinical diagnosis was of an atypical melanocytic lesion.

The presence within the lesion of a dermal nevus made the dermoscopic diagnosis even more difficult. In fact, the overall dermoscopic appearance of our lesion revealed the 4 most important features of malignant lentigo in progression: asymmetric pigmented follicular openings, dark rhomboidal structures, slate-gray globules, and slate-gray dots.

This case shows the difficult clinical and dermoscopic differential diagnosis of pigmented lesions on the face, mostly with darker skins, and shows that it is important to consider also lichenoid dermatitis as a possible differential diagnosis in the management of pigmented lesions of the face.
